# Knowledge, Attitude, and Preventive Practice Related to Onchocerciasis and Associated Factors among Resource-Limited Selamogo Woreda Residents, South West Ethiopia, 2021

**DOI:** 10.1155/2022/2481841

**Published:** 2022-08-29

**Authors:** Girma Gilano, Mulugeta shegaze, Amanuel Dubale

**Affiliations:** ^1^Department of Health Informatics, School of Public Health, College of Medicine and Health Sciences, Arba Minch University, Arba Minch, Ethiopia; ^2^Department of Public Health, School of Public Health, College of Medicine and Health Sciences, Arba Minch University, Arba Minch, Ethiopia; ^3^Department of Medical Laboratory, School of Medicine, College of Medicine and Health Sciences, Arba Minch University, Arba Minch, Ethiopia

## Abstract

**Introduction:**

Ethiopians work very hard to control and eradicate the vector and the parasite of Onchocerciasis. However, some hard-to-reach areas are not adequately covered by interventions that have previously taken place in various endemic sites in the country. This study aimed to assess knowledge, attitude, and preventive practice of Onchocerciasis among Selamogo residents to enhance the expansion of interventions.

**Methods:**

We used a survey questionnaire to capture the data on 572 Selamago residents. We checked, cleaned, entered the data into EPI Info v.7, and analyzed it in STATA v.15. We fitted a binary logistic regression model to examine the associated factors. Variables significant at *P* < 0.20 were included in the model. We declared association at *P* < 0.05.

**Results:**

Of the 578 residents we approached, 572 (99%) responded to the interview. We found 48.30% of poor knowledge, 90.90% of poor attitude, and 85.70% preventive practice related to Onchocerciasis. Factors like Amhara ethnicity (AOR = 2.35, 95% CI: 1.05, 5.27), Orthodox Christianity (AOR = 1.87, 95% CI: 1.12, 3.10), Muslims (AOR = 2.28, 95% CI: 1.05, 4.94), secondary school (AOR = 2.31, 95% CI: 1.50, 3.55), diploma (AOR = 10.34, 95% CI: 4.62, 23.16), and preventability of Onchocerciasis (AOR = 3.02, 95% CI: 1.39, 6.55) were associated with KAP. Other factors like medical treatability, admission history to the health facilities, sex, and the number of households were also associated.

**Conclusion:**

The KAP related to Onchocerciasis is very poor compared to the indicators and evidence in the country. An intervention that considers local resources like taking the experience of groups with good knowledge, attitude, and preventive practice led by educated plus positive attitude people regarding the preventability and treatability of Onchocerciasis might be mandatory to improve the status.

## 1. Introduction

Neglected Tropical Diseases (NTDs) are the common cause of illness in low-income populations of Africa, Asia, and the South Americas. They are very common in 31 sub-Saharan African countries [1, 2]. Onchocerciasis is a parasitic disease that causes ocular and nodular lesions in humans, and the transmission is mediated by the vector blackflies [3, 4]. According to the 1995 WHO report, 17.5 million Africans were affected, followed by 140,000 Americans and 30,000 people in the Arabic peninsula, with 267,950 cases of blindness [4–6]. According to the African Program for Onchocerciasis Control (APOC), 99% of blindness is in Africa [7]. From 1974 to 2002, 11 West African countries successfully managed to control Onchocerciasis with the help of the Onchocerciasis Control Program (OCP) [8]. Nigeria in 2012 [9], Guatemala in 2016, Colombia in 2013, Ecuador in 2014, and Mexico in 2015 [10] reported zero cases. In sub-Saharan countries, the number of people treated for microfilaria, the number of people protected from the parasite, and areas freed from infection have increased in the last 30 years [11]. In East Africa, a program focused on Southwestern Uganda reported the disappearance of microfilaria in 2012 [12]. Kenya eliminated vectors in the 1960s without treatment [13]. In Ethiopia, the 2017 status review report showed Onchocerciasis is endemic in 188 districts [14]. One report showed that the drastic environmental change caused the disappearance of the parasite in the Tigray region [15].

In developing countries like Ethiopia, knowledge, attitude, and preventive practice are important in communities to control and eradicate Onchocerciasis [16]. The first-ever epidemiological assessment in Ethiopia was done in the 1970s in Illubabor, where the prevalence rate of Onchocerciasis was 51.4% and 66.7% in two areas [17]. Like all African countries, Ethiopia has a plan of eradicating Onchocerciasis by 2030 through CDTI, vector control, and other methods [18]. In one study in Southwest Ethiopia, 64.4% of participants had the disease but only 36.5% thought Onchocerciasis was transmissible [19]. Nigeria, Senegal, and Mali eliminated Onchocerciasis only by ivermectin intervention at least in some parts of their countries [9]. The ivermectin strategy outways the vector controlling mechanism that is complicated by the long-living worms in human skin (0–15 years). There are even many programs (OCP, APOC, and CDTI) trying to control Onchocerciasis, which are successful in West Africa. The OCP is specially confined to the region [20]. The knowledge of the disease, attitude, and preventive practices have been very important parameters for community campaigns to control Onchocerciasis and to bring the matter to the community [21]. In North West Ethiopia, the misconceptions regarding the disease and the poor knowledge, attitude, and practice were identified as obstacles [16]. In other words, the status review showed that community readiness comes after the direct increase in community awareness, attitude, and preventive practices [14]. For the 2025 plan of Onchocerciasis eradication, community participation is forwarded as the crucial strategy in Africa [22]. Without understanding experience in every hard-to-reach area of the countries, improving knowledge, attitude, and preventive practice regarding Onchocerciasis might be impossible to facilitate interventions. The study in the Bench Maji zone showed that many years of treatment with ivermectin did not reduce the prevalence of the disease in the area, which might be a good indication of the necessity of improving knowledge, attitude, and practice [23, 24]. There was no status evidence and no other studies have ever been conducted on Onchocerciasis in the current area despite its endemic nature. Thus, this study aimed at assessing knowledge, attitude, preventive practice, and associated factors in Selamago woreda to uplift the attention of stakeholders and policymakers.

## 2. Materials and Methods

### 2.1. Study Design and Setting

The community-based cross-sectional study design will be employed in the South Omo zone, Selamgo Woreda, from March 2021 to January 2022.

South Omo Zone is one of the 15 zonal administrations of Southern Ethiopia. **Selamago** is one of the woredas in the zone. As a part of the Debub Omo Zone, Selamago is bordered on the south by Nyangatom on the west and north by the Omo River, which separates it from the Bench Maji, Keffa, and Konta. In the northeast, it is bordered by the Gamo Gofa, on the east by the Basketo and Bako Gazer, and on the southeast by the Usno River, which separates it from Bena Tsemay. The Mago River defines a part of the boundary with Bako Gazer. The administrative center of Selamago is in Hana. David Turton describes this area as one of the most isolated in Ethiopia. The Omo and Mago rivers make access difficult, and the conquering armies of Menelik II bypassed them. Although the occupying Italians briefly occupied a military post along the Omo in what later became Selamago in 1940, it was not until the 1970s that direct Ethiopian administration reached this area [25].

### 2.2. Participants

We selected 6 kebeles (smallest administrative level) from a possible 14, and households from the randomly selected kebeles' were randomly included. All individuals in the randomly included households during the data collection period whose age was ≥ 18 years old and who has been a resident for at least 6 months were eligible for a face-to-face interview. We excluded residents who were physically or mentally unable to communicate with the interviewer and when there were no other possibilities of obtaining their information.

### 2.3. Sample Size Determination and Sampling Procedures

We calculated the sample size using the single population proportion formula. The prevalence rate of knowledge, attitude, and practice is considered 50% since no previous study has been obtained in this specific area. Considering 95% CI, a 5% margin of error, and adding a 10% of nonresponse rate, the final sample size calculated using EPI Info 7 was 578. To achieve the selection, we used systematic sampling methods. We randomly selected kebeles and proportionally allocated samples to each kebele using sample fractions depending on the population of each kebele. We obtained individual households by dividing the number of households by sample size as 2000/578 = 4 : 1. Using the lottery method, the first household from 1 to 4 was drawn, and every 4^th^ household that fulfilled the inclusion criteria for the interview was chosen.

### 2.4. Variables of the Study

The outcome variables of this study were knowledge, attitude, and preventive practices. To address the knowledge, 6 item questions regarding clinical manifestations, organs affected, cause, and transmission of the disease were considered. All items were measured through yes/no. Yes (present, known, accepted…) scored as “1” and No (do not know, not agree, not true or others) scored as “0”. The combination of the items' scores becomes “1” to mean good and “0” to mean poor knowledge.

To address information on attitude, 7 parameters were prepared regarding the presence/absence of stigmatization and the preferable place for treatments and others to indicate a good or poor/low attitude. All items were measured using yes/no. Positive attitude responses were categorized as good/positive attitude [1] and poor/negative or low attitude (0). We combined as we did under knowledge. Prevention practices were collected using three questions “When a member of your family suffers from Onchocerciasis, what did she/he use to treat the disease?”, “Where did he/she seek help drug/others,” and “do you use preventive methods to avoid Onchocerciasis” with the responses drug/other, HC/Hospital/others, and yes/no, respectively. The correct answer was coded “1” which is good practice, and “0” represented the incorrect answer as poor/low practice [21]. In some of the questions like where to seek help, the answer can be health center (HC), or hospital, and others; however, we combine 1 and 2 as 1 and others as 0. This is similar in both practice and attitude measurements. For all three correct answers, more than half the score was taken as good.

The independent variables of the study were socio-demographic information including age (number), gender(male/female), occupation(government employee,, farmer, housewife, merchant, daily laborer, or others), number of households(number), and educational status of respondents(illiterate, primary school, secondary school,/preparatory, diploma, 1^st^ degree and above), living kebele, medical treatability of the disease(yes/no), admission (yes/no), Onchocerciasis preventability (yes/no), socio-economic (number), presence of chronic illness(yes/no), marital status(single, married, divorced, separated, and widowed), religion(orthodox, Muslims, Protestant, Catholic and others), and ethnicity(Dime, Bodi, Mursi, Amhara, and others).

### 2.5. Data Collection Instruments and Data Quality

The instrument for data collection was designed after a vigorous literature review in the disease domain. It contains 6 knowledge-based questions, 7 questions about attitudes, and three questions on preventive practice along with socio-demographic variables [21].

Twelve (diploma) trained data collectors who know the local language participated based on their previous experience in data collection and research. We assigned three supervisors (MSc) to follow the process and handle the fieldwork. The questionnaire was translated to Amharic and back to English to see the consistency. Before data collection, the tool was tested on 5% of the population in far unselected kebeles. Issues related to understanding, language, and consistency during completing questionnaires were corrected and included in the final questionnaire.

### 2.6. Data Processing and Analyses

After completion of the field checkup, the principal investigators received, checked, and entered the data into EPI Info version 7. We used binary logistic regression to examine the factors that affected knowledge, attitude, and practice. All analyses were performed in STATA 15. *P* value <0.20 was used to include variables in the model and *P* value <0.05 was used to declare association. We presented descriptive statistics using numbers, means, standard deviation (±SD), and percentages. Using 95% CI and adjusted odds ratio, we presented association data.

### 2.7. Ethical Consideration

Primarily, we obtained ethical clearance from the Institutional Review Board of Arba Minch University with issue number AMU-IRB/1123/2021, and then the permission letters were collected from the Zonal Health Departments and Selamago woreda health office. Verbally, consent was secured from each participant, and the right to refuse or participate was granted and respected. The information obtained from the study was kept confidential.

## 3. Results

### 3.1. Descriptive Statistics

**Figure 1:**
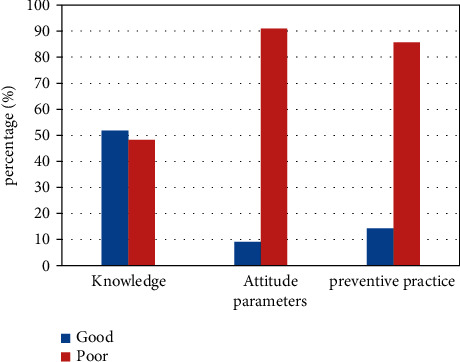
The knowledge, attitude, and preventive practice related to Onchocerciasis among Selamago woreda residents in 2021.

Of 578 residents approached, 572 (99%) responded to the interview. We found 48.30% of poor knowledge, 90.90% of poor attitude, and 85.70% of poor preventive practice related to Onchocerciasis among Selamago woreda residents. Descriptive statistics indicate that participants had an average household income of 698.53 ± 92.9, a mean age of 37.55 ± 8.25, an average number of children of 4.02 ± 1.56, and a mean household size of 5.98 ± 1.70. The largest ethnic group was Dime (45.10%), followed by Bodi (27.40%). The largest proportion of the participants was illiterate (54.90%), and farmers accounted for 55.80% of the total followed by pastoralists at 36.20%. The largest proportion of participants (92.70%) believe Onchocerciasis is unpreventable and 71.00% believe Onchocerciasis is medically untreatable (Table 1 and Figure 1).

### 3.2. Analysis of Factors Associated with Knowledge, Attitude, and Preventive Practice of Onchocerciasis

Examination of socio-demographic and other factors showed that ethnicity, religion, education status, number of households, the experience of medical treatability of the disease, and preventability were associated with knowledge of Onchocerciasis. Accordingly, Amhara's ethnic background was associated with 2.35 times increased knowledge related to Onchocerciasis with AOR of 2.35(1.05, 5.27). The odds of good knowledge were higher among Orthodox Christian and Muslim religions with AOR of 1.87(1.12, 3.10) and 2.28(1.05, 4.94), respectively, compared to protestant religion followers. Good knowledge was also observed among participants who learned (secondary school 2.31(1.50, 3.55), diploma 10.34(4.62, 23.16), and first degree or above 8.95(3.97, 20.21)) compared to illiterate participants. Participants from larger households had good knowledge of Onchocerciasis with an AOR of 1.15(1.03, 1.30); similarly, the odds of good knowledge were higher among participants regarding preventability of Onchocerciasis with AOR of 3.02(1.39, 6.55) (Table 2).

The odds of a good attitude were higher among female participants with an AOR of 2.17(1.04, 4.56). The ethnic groups of Mursi and Amhara were associated with higher odds of good attitude with AOR of 3.74(1.47, 9.52) and 5.31(1.18, 23.85) respectively. Participants at primary level education had higher odds of good attitude with AOR of 3.68(1.38, 9.80) compared to illiterates. Participants who believe Onchocerciasis is medically treatable had higher odds of good attitude with AOR of 2.80(1.24, 6.31). However, participants with previous admission history had a lower attitude related to Onchocerciasis with AOR of 0.39(0.16, 0.93) (Table 3).

In this study, 70% of females were not practicing preventive measures for Onchocerciasis with AOR of 0.30(0.10, 0.90). The participants from the Mursi ethnicity had 77% reduced preventive practice related to Onchocerciasis with AOR of 0.23(0.08, 0.72); while, ethnic Amhara had higher odds of good preventive practice with AOR of 5.23(1.13, 24.23). Orthodox Christianity was also associated with the higher odds of the preventive practice of Onchocerciasis with AOR of 4.67(1.32, 16.57). The participants who believe Onchocerciasis is medically treatable had 53% reduced odds of preventive practice with AOR of 0.47(0.24, 0.92), the previous admission was associated with good Onchocerciasis preventive practice with AOR of 2.25(1.13, 4.50) (Table 4).

## 4. Discussion

From the analysis of the data, we found 48.30% of poor knowledge, 90.90% of poor attitude, and 85.70% poor preventive practice related to Onchocerciasis among Selamago woreda residents. This is less than the finding from the study conducted in South-Western Ethiopia which showed poor knowledge of 33%, poor attitude of 36%, and poor practice of 39.6% [19]. The inconsistency might be associated with the hard-to-reach nature of the current area causing the gap. The area was out of road access from both the federal and the regional government before the recent opening of the sugar factory, which came with the opportunity. The pastoralist lifestyle, accessibility of services, and availability of Onchocerciasis-related information might be the reason that both regional and federal governments are unable to reach the area. Only 7.30% of the respondents think Onchocerciasis is a preventable disease. Another study in Southwest Ethiopia showed that 88.2% of the respondents perceived Onchocerciasis as preventable [26]. Furthermore, 93.3% of participants from the study conducted in Northwest Ethiopia believed that Onchocerciasis is preventable [16]. The lower perception of preventability of Onchocerciasis might be due to the socio-demographic differences and poor access to health education information. In other words, 71% of respondents believed Onchocerciasis is medically untreatable. From other studies, 94.0% of participants think Onchocerciasis is treatable [22 ] and 90.3% of participants from another study also think Onchocerciasis is treatable [19]. The current study area is one of the hard-to-reach areas in the country. This might limit access to and from resourceful areas and cause poor socio-economic development. More than half (54%) of the participants were illiterate. The study in the Southwest indicated that 21.8% of the participants were illiterate [19]. This might show how the current study area is lagging in basic socio-demographic factors. The poor educational achievement might be the cause for other poor achievements in other socio-demographic factors. In every aspect, the targets to eliminate Onchocerciasis by 2025 and 2030 might be in big trouble, as reaching them will be impossible without sufficiently covering such hard-to-reach areas [27, 28].

Participants with larger households showed good knowledge related to Onchocerciasis; however, there is no evidence to support this information from previous studies. In other words, people who had good knowledge do not exactly think Onchocerciasis is preventable. The finding is supported by other studies [16, 26]. The consistency might be due to the lack of objective knowledge obtained from credible sources to rely on. It might indicate, the poor objective knowledge regarding the disease in the community. Educational achievement was associated with both good knowledge and attitude related to the disease. This is supported by many previous studies [19, 23, 29, 30]. The consistence might indicate that educational status is an independent predictor of knowledge and attitude related to Onchocerciasis, which is a good opportunity for further intervention. Both ethnic groups Mursi and Amhara had good knowledge and attitude; however, ethic Mursi had poor preventive practice [16]. This might show that local strategy to increase KAP toward Onchocerciasis is possible through using rich experiences among different ethnic groups. Similarly, female participants showed a good attitude but practically males were great. This is also supported by another study in the country [16]. Sex is one of the factors that cause prevalence differences in groups in Ethiopia [24]. This consistent information shows that sex is another persistent predictor to consider during interventions. The knowledge and attitude toward medical treatability of Onchocerciasis are well associated with the knowledge and attitude of the participants; however, in practice, it is poor. From other studies, a large proportion of respondents believe Onchocerciasis is a preventable disease [16,19]. This might indicate that even though knowledge and attitudes might be good, the need for improving practice might require another gear. Additionally, participants with a history of previous admission had poor attitudes but had good preventive practices for Onchocerciasis [31]. This might mean that having experience in service taking can improve attitude but practice might need objective intervention. In other words, the Orthodox Christian religion was associated with both good knowledge and practice. This is not supported by the study in the Bench Maji zone [23]. A small group of people who mostly migrated from other places practice the orthodox religion and they might have experience and practice they acquired probably from other areas. This also shows that local resources to reach other groups are available in the woreda. Despite all the important findings, the study has also some limitations. We used a questionnaire for all dependent variables that might not be good to assess practice, experience, and educated from the same area for data collection. We picked questions that can easily assess practice and attitude without the requirement of observation and data collectors from each Kebele which helped us to achieve a full capture of information.

## 5. Conclusion

The knowledge, attitude, and preventive practice related to Onchocerciasis are very poor compared to indicators and other evidence. The target Elimination of Onchocerciasis by 2025 in Africa might not be achieved in such hard-to-reach areas in Ethiopia. Factors like sex, ethnicity, educational status, religion, and medical treatability of Onchocerciasis were associated with the KAP. An intervention that considers local resources like the experience of groups, attitude, and preventive practice led by educated people who have positive attitudes regarding preventability and treatability of Onchocerciasis might be mandatory to improve the current status. The support of religious leaders, a community committee with a sufficient number of female members, and other stakeholders might be vital to the success of the efforts.

## Figures and Tables

**Table 1:** The distribution of socio-demographic characteristics of the study participants at Selamago woreda in 2021.

Variables	N (%)
Sex	
Male	213 (37.20)
Female	359 (62.80)

Ethnicity	
Dime	258 (45.10)
Bodi	157 (27.40)
Mursi	106 (18.50)
Amhara	51 (8.90)

Marital status	
Single	175 (30.6)
Married	387 (67.70)
Divorced	10 (1.70)

Educational status	
Illiterate	314 (54.90)
Primary school	26 (4.20)
Secondary/preparatory school	132 (23.10)
Diploma	54 (9.40)
First degree and above	48 (8.40)

Preventability of Onchocerciasis	
No	530 (92.70)
Yes	42 (7.30)

Medically treatability	
No	406 (71.00)
Yes	166 (29.00)

Religion	
Protestant	157 (27.40)
Orthodox	387 (67.70)
Muslims	10 (1.70)

Occupation	
Farmer	319 (55.80)
Housewife	37 (6.50)
Daily laborer	9 (1.60)
Pastoralists	207 (36.20)

**Table 2:** Distribution of factors associated with knowledge related to Onchocerciasis in Selamago in 2021.

Variable	*P* value	AOR	95% CI	
			Lower	Upper
Ethnicity				
Dime	1.00			
Bodi	0.11	1.44	0.91	2.29
Mursi	0.24	0.61	0.27	1.37
Amhara	0.03	2.35	1.05	5.27

Religion				
Protestant	1.00			
Orthodox	0.01	1.87	1.12	3.10
Muslims	0.03	2.28	1.05	4.94

Educational status				
Illiterate	1.00			
Primary school	0.33	1.52	0.64	3.61
Secondary/preparatory school	0.001	2.31	1.50	3.55
Diploma	0.001	10.34	4.62	23.16
First degree and above	0.001	8.95	3.97	20.21
Number of households	0.014	1.15	1.03	1.30

Medically treatable				
No	1.00			
Yes	0.005	3.02	1.39	6.55

Onchocerciasis is preventable				
No	1.00			
Yes	0.005	0.57	0.39	0.84

**Table 3:** Distribution of factors associated with attitude related to Onchocerciasis in Selamago in 2021.

Variables	*P* value	AOR	95% CI	
			Lower	Upper
Sex				
Male	1.00			
Female	0.03	2.17	1.04	4.56

Ethnicity				
Dime	1.00			
Bodi	0.61	1.42	0.37	5.39
Mursi	0.006	3.74	1.47	9.52
Amhara	0.03	5.31	1.18	23.85

Educational status				
Illiterate	1.00			
Primary school	0.009	3.68	1.38	9.80
Secondary/preparatory school	0.54	0.79	0.37	1.68
Diploma	0.21	3.78	0.47	30.74
First degree and above	0.41	2.62	0.26	26.45

Medically treatable				
No	1.00			
Yes	0.01	2.80	1.24	6.31

Ever admitted to a health facility				
No	1.00			
Yes	0.03	0.39	0.164	0.93

**Table 4:** Distribution of factors associated with preventive practice related to Onchocerciasis in Selamago in 2021.

Variables	*P* value	AOR	95% CI	
			Lower	Upper
Sex				
Male	1.00			
Female	0.03	0.30	0.10	0.90

Ethnicity				
Dime	1.00			
Bodi	0.08	1.73	0.94	3.18
Mursi	0.01	0.23	0.08	0.72
Amhara	0.03	5.23	1.13	24.23

Religion				
Protestant	1.00			
Orthodox	0.02	4.67	1.32	16.57
Muslims	0.08	2.74	0.88	8.54

Medically treatable				
No	1.00			
Yes	0.03	0.47	0.24	0.92

Ever admitted to health facility				
No	1.00			
Yes	0.02	2.25	1.13	4.50

## Data Availability

The data used in this study are available unrestrictedly and can be supplied with the final submission of the accepted manuscript.

## Abbreviations

KAP: Knowledge, attitude, and practice
AOR: Adjusted odds ratio
CI: Confidence interval
CDTI: Community directed treatment with ivermectin
UNDP: United Nations Development Program
OCP: Onchocerciasis control program
APOC: African Program for Onchocerciasis Control.
